# Regulatory variants of *APOBEC3* genes potentially associate with COVID-19 severity in populations with African ancestry

**DOI:** 10.1038/s41598-023-49791-x

**Published:** 2023-12-17

**Authors:** Ke Zhang, Fang Chen, Hu-Yan Shen, Ping-Ping Zhang, Han Gao, Hong Peng, Yu-Si Luo, Zhong-Shan Cheng

**Affiliations:** 1 The Key and Characteristic Laboratory of Modern Pathogenicity Biology, School of Basic Medical Sciences, Guizhou Medical University, Guiyang, 561113 China; 2 The Department of Emergency ICU, The Affiliated Hospital of Guizhou Medical University, Guiyang, 550004 China; 3 The Department of Emergency, Liupanshui Hospital of The Affiliated Hospital of Guizhou Medical University, Liupanshui, 553000 China; 4 Center for Applied Bioinformatics, St. Jude Children’s Research Hospital, 262 Danny Thomas Hospital, MS1122, Memphis, TN 38105 USA

**Keywords:** Genetics, Risk factors

## Abstract

Since November 2019, the severe acute respiratory syndrome coronavirus 2 (SARS-CoV-2), has caused the worldwide pandemic of the coronavirus disease 2019 (COVID-19), the impact of which is huge to the lives of world populations. Many studies suggested that such situation will continue due to the endless mutations in SARS-CoV-2 genome that result in complexity of the efforts for the control of SARS-CoV-2, since the special enrichment of nucleotide substitution C>U in SARS-CoV-2 sequences were discovered mainly due to the editing by human host factors *APOBEC3* genes. The observation of SARS-CoV-2 variants Beta (B.1.351) and Omicron (B.1.1.529) firstly spreading in South Africa promoted us to hypothesize that genetic variants of *APOBEC3* special in African populations may be attributed to the higher mutation rate of SARS-CoV-2 variants in Africa. Current study was conducted to search for functional variants of *APOBEC3* genes associate with COVID-19 hospitalization in African population. By integrating data from the 1000 Genomes Project, Genotype-Tissue Expression (GTEx), and Host Genetics Initiative (HGI) of COVID-19, we identified potential functional SNPs close to *APOBEC3* genes that are associated with COVID-19 hospitalization in African but not with other populations. Our study provides new insights on the potential contribution of *APOBEC3* genes on the evolution of SARS-CoV-2 mutations in African population, but further replication is needed to confirm our results.

## Introduction

Since November 2019, the emergence of severe acute respiratory syndrome coronavirus 2 (SARS-CoV-2) has resulted in coronavirus disease 2019 (COVID-19), affecting everything from daily routines to mental wellbeing on global populations. Three years into the pandemic, SARS-CoV-2 continues to have a profound effect on human health, such as the emergence of a new type of disease: long COVID, which is characterized by a diverse range of symptoms, including over 200 symptoms that have been reported so far^1^. Study suggests that the ongoing mutations in the genome of SARS-CoV-2 contribute to the complexity of controlling the pandemic by false-negative result in viral RNA genome sequencing^2^, with the 12 types of nucleotide substitutions reported. It is noteworthy that the C>U mutation among the 12 types of nucleotide substitutions occurs much more frequently than other types^3^.

Regarding previously discovered viral mutations of human immunodeficiency virus (HIV) and monkeypox virus (MPOXV), the potential role of non-random driver proteins, such as Apolipoprotein B mRNA-editing catalytic polypeptide-like 3 (APOBEC3) was assessed and analyzed by researchers^4^. These APOBEC3 proteins share a highly conserved zinc-dependent deaminase domain that is activated by a zinc ion that coordinates a water molecule for nucleophilic attack of C4 of the pyrimidine ring of C, which in conjunction with the glutamic acid residue leads to the deamination of the base to U^2^. Therefore, the hypermutation events of C>U take place in viral genome during subsequent cellular infection^5,6^. Furthermore, the APOBEC3 family comprises cytidine deaminases that contribute to the innate response against retroviral and retrotransposon infection^7^. They use deaminase and deaminase-independent mechanisms to suppress various endogenous and exogenous viruses^8^.

Among seven members in the APOBEC3 family, at least five of these enzymes, namely APOBEC3B, APOBEC3D, APOBEC3F, APOBEC3G, and stable haplotypes of APOBEC3H, exhibit anti-HIV activity^9^. As one of the potent mutators in the human genome, APOBEC3 stands out as the only one whose expression is significantly upregulated after HIV infection^10^. In recent years, MPOXV, a double-stranded DNA virus, has also undergone a surprising surge in mutation and was declared a Public Health Emergency of International Concern by WHO in 2022. A curious observation is that most cases of HIV and original strains of MPOXV^11^ were reported in Africa. Notably, the SARS-CoV-2 variants Beta (B.1.351) and Omicron (B.1.1.529) initially emerged in South Africa and eventually spread to other countries^12^. Furthermore, the pivotal mutation such as D614G in the Spike protein that is caused by the C>U mutation in SARS-CoV-2 genome, was identified in almost all Beta (B.1.351) and Omicron (B.1.1.529) isolates, but not in Alpha (B.1.1.7), Gamma (P.1), and Delta (B.1.617.2) strains^13^. This suggests that the beta and omicron variants may have contributed a higher number of key mutations to the global COVID-19 pandemic. All of these observations indicate that the African population may differ from other populations in their role in driving viral mutations, whether in the context of SARS-CoV-2 or other viruses. One potential explanation for the prevalence of the SARS-CoV-2 variants in Africa is the low vaccination rate on the continent, which might increase the likelihood of virus transmission, subsequently leading to a higher probability of mutagenesis with each replication cycle and the emergence of multiple variants^14^. However, despite the vaccine rollout, morbidity and mortality rates have remained low in Africa. This cannot be adequately accounted for by the younger age of the African population alone, suggesting the potential involvement of genetic factors in COVID-19 susceptibility or severity^15^. Thus, the current study was conducted to investigate whether functional genetic variants of *APOBEC3* genes associate with COVID-19 severity in African populations.

## Materials and methods

### Association of single nucleotide polymorphisms (SNPs) of *APOBEC3* genes with COVID-19 hospitalization

We firstly downloaded the summary statistics of COVID-19 hospitalization genome-wide association studies (GWASs) conducted separately among samples with European or African ancestries from the COVID-19 Host Genetics Initiative (HGI, release 7)^16,17^. The links to the two GWAS summary statistics are freely available at HGI: (1) HGI-B2-EUR GWAS of hospitalized COVID-19 vs. general population controls among European samples (https://storage.googleapis.com/covid19-hg-public/freeze_7/results/20220403/pop_spec/sumstats/COVID19_HGI_B2_ALL_eur_leave23andme_20220403.tsv.gz; reference in hg38 build) and (2) HGI-2-AFR GWAS of hospitalized COVID-19 vs. general population controls with African ancestry (https://storage.googleapis.com/covid19-hg-public/freeze_7/results/20220403/main/sumstats/COVID19_HGI_B2_ALL_leave_23andme_20220403.tsv.gz; reference in hg38 build). The sample sizes for both GWASs are as follows: HGI-B2-EUR (cases = 32,519 and controls = 2,062,805) and HGI-B2-AFR (cases = 2,589 and controls = 123,225). Their summary statistics were undertaken standard quality controls by HGI, and we thus used the data directly in our analyses for *APOBEC3* genes. The association signals around the *APOBEC3* genes, including *APOBEC3A*, *APOBEC3B*, *APOBEC3C*, *APOBEC3D*, *APOBEC3F*, *APOBEC3G*, and *APOBEC3H*, are located in a gene cluster on chromosome 22 (chr22:38, 939, 327–39, 106, 168; hg38). Therefore, the COVID-19 association signals for the *APOBEC3* genes in the above two GWASs were extracted, with only top SNPs passed the association *P* < 0.01 were selected for downstream functional evaluation. The use of relaxed association threshold was arbitrarily set since we thought that our study was a typical candidate gene study.

### Minor allele frequencies for prioritized *APOBEC3* genes across populations from the 1000 genome project

For 6 candidate SNPs passed the nominal association threshold *P* < 0.01, we determined their minor allele frequencies across multiple major populations, including African (AFR), Ad Mixed American (AMR), East Asian (EAS), European (EUR), and South Asian (SAS), as well as the corresponding subpopulations for these major populations from Ensembl database^18^. Ensembl curated the population frequency data from the Phase 3 of the 1000 Genome Projects^19^. The module “Population genetics” of Ensembl was used to extract the allele frequencies of 6 target *APOBEC3* SNPs.

### cis-expression quantitative trait locus (cis-eQTL) analysis of *APOBEC3* SNPs in Genotype-Tissue Expression (GTEx) database

For these top SNPs of *APOBEC3* genes that passed the COVID-19 association threshold *P* < 0.01, we annotated them individually with Haploreg4^20^ and the GTEx V8^21^. As both Haploreg4 and GTEx provide expression quantitative trait locus (eQTL) information for these SNPs or its high linkage disequilibrium (LD) SNPs (R^2^ > 0.8), we curated all eQTL information for these top SNPs of *APOBEC3* genes. When a SNP was not included by the two databases, we obtained one of its high LD SNPs via Haploreg4 in European or African population dependent on whether the SNP was emerged from HGI-B2-EUR or HGI-B2-AFR GWAS, and then searched for the selected high LD SNPs in the two databases.

### *APOBEC3* gene expression analysis between European American (EA) and African American (AA) in GTEx database

The GTEx database encompasses the gene expression data in 49 tissues for individuals with multiple ancestries, such as European ancestry, African ancestry, as well as Asian ancestry. Recently, a study published by Gay N.R. et al.^22^ estimated the ancestries of GTEx samples, the results of which are openly accessible. According to Gay N.R. et al.^22^, among 838 GTEx individuals, there are 103 individuals with African ancestry, and most of others (n = 715) are EA. Based on these EA and AA sample identification labels provided by Gay N.R. et al., we mapped RNA-seq gene expression data downloaded from GTEx of each sample to its corresponding ancestry by looking up with the sample names from GTEx and Gay N.R. et al.^22^. The GTEx TPM (transcripts per million) matrix was downloaded from this link: https://storage.googleapis.com/gtex_analysis_v8/rna_seq_data/GTEx_Analysis_2017-06-05_v8_RNASeQCv1.1.9_gene_tpm.gct.gz. We thus conducted differential gene expression analysis by stratifying *APOBEC3* genes expression by ancestry among 49 GTEx tissues. The multiple testing adjusted statistical significance was set at *P* < 1.5 × 10^–4^ [i.e., 0.05/(7 × 49)] for differential gene expression analysis between EA and AA samples using SAS statement “lsmeans”, which is included in the SAS procedure “proc GLM” via the freely available SAS OnDemand for Academics.

### *APOBEC3* gene expression among blood samples derived from healthy controls and COVID-19 patients

These 7 *APOBEC3* genes were subjected to differential gene expression analysis in blood samples of COVID-19 patients and healthy controls published by Thair et al.^23^ using the COVID19db, a gene expression database related to SARS-CoV-2 infection^24^. Statistical significance was determined by one-way ANOVA, with the multiple testing significance threshold set at *P* < 0.01 = 0.05/7*.*

### Analysis of *APOBEC3* induced C>U coding mutation among ~ 8.4 million SARS-CoV-2 genomes from African, Europe, and North America

Mutational data from SARS-CoV-2 genomes were obtained from the open database shared by nextstrain (https://nextstrain.org/ncov/open/global/6m). SARS-CoV-2 coding mutations were identified by nextstrain using Wuhan-Hu-1/2019 (GenBank accession number MN908947) as the reference genome. To quantify the total number of C>U mutations in coding regions and the overall number of coding mutations in each SARS-CoV-2 genome within the downloaded mutational metadata (https://data.nextstrain.org/files/ncov/open/metadata.tsv.zst), we developed a customized Perl script. And our analysis retained only SARS-CoV-2 genomes that met two criteria: they had at least 10 coding mutations, and they were sampled from three different geographic regions, including Africa, Europe, and North America. The percentage of C>U coding mutations relative to the total number of coding mutations (referred to as the "C>U coding mutation percentage") in each SARS-CoV-2 genome was determined using SAS OnDemand for Academics. This software was also used to generate histograms illustrating the distribution of C>U coding mutation percentages across the three geographic regions. Furthermore, given that the average C>U coding mutation percentage is approximately 40% across all genomes from these three geographic regions (comprising around 8.4 million viral genomes), we further calculated the percentage of SARS-CoV-2 genomes exhibiting a higher C>U coding mutation percentage. We stratified SARS-CoV-2 samples within each geographic region based on a C>U coding mutation percentage threshold of 40%. Finally, we employed a Chi-square test to assess differences in the percentage of SARS-CoV-2 genomes with higher C>U coding mutation percentages between the African samples and the samples from the other two regions.

### Issue of medical ethics

As the two GWAS summary statistics are freely available at HGI and no patients’ identification information were revealed by HGI, our study was thus a typical secondary analysis of previously published data. In addition, we didn’t generate any new data in our study. All data published by other researchers were further processed to investigate the potential genetic variants of *APOBEC3* genes associated with COVID-19 hospitalization. Based on this, we believed that our study did not involve any issues of medical ethics and no committee permission is applicable to our study. Finally, we confirm that all methods were carried out in accordance with relevant guidelines and regulations in our universities or institutes, and no experiments were performed with human subjects in our study, thus we emphasize that no approval and consent forms are required for our investigation.

## Results

**Figure 1:**
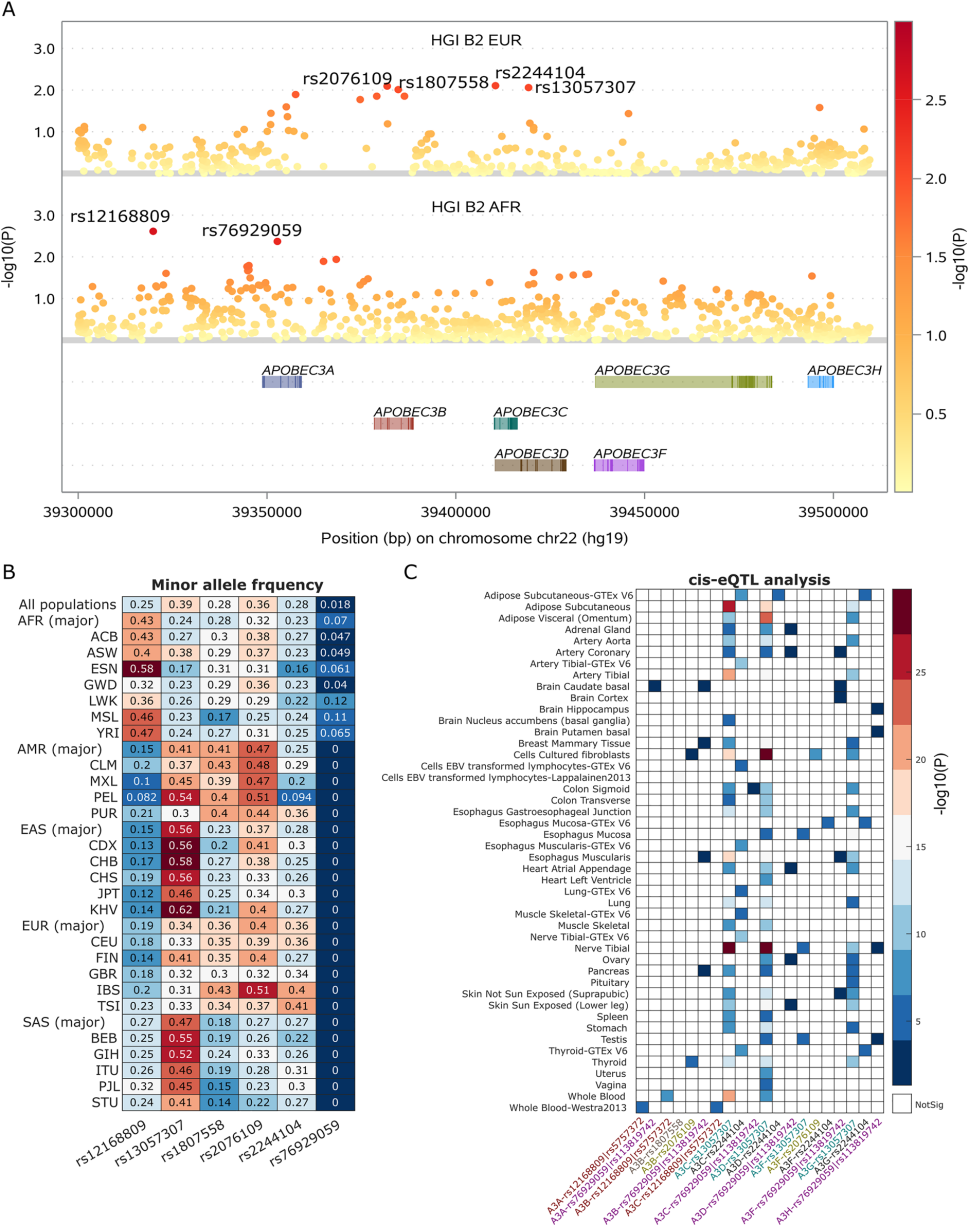
Association of single nucleotide polymorphisms (SNPs) of *APOBEC3* genes with COVID-19 hospitalization. (**A**) Local Manhattan plots demonstrate that 6 SNPs close to *APOBEC3* genes display nominally association signals (*P* < 0.01) in two COVID-19 hospitalization GWASs with different ancestries, including HGI-B2 of samples with European or African ancestries. Among these 6 COVID-19 risk SNPs, 2 SNPs are specific derived from the COVID-19 hospitalization GWAS with African ancestry and the other 4 SNPs are unique to the COVID-19 hospitalization GWAS with European ancestry. (**B**) Population frequencies of these 6 SNPs across 5 major populations, including African (AFR), Ad Mixed American (AMR), East Asian (EAS), South Asian (SAS), and European (EUR), as well as among all or sub-populations (full annotations included in Table S1) of these major populations are illustrated in a heatmap. Each heatmap cell displays the population frequency of each corresponding SNP. The AFR-specific COVID-19 risk SNP rs76929059 is only polymorphic in AFR populations. (**C**) cis-eQTL analysis for these 6 COVID-19 risk SNPs or its high linkage disequilibrium (R^2^ > 0.9 in EUR or AFR populations) across multiple tissues in GTEx and other eQTL databases. These tissues display cis-expression quantitative trait locus (cis-eQTL) signals are mainly from GTEx V8, the label of which are omitted to save space in the figure.

To investigate potential functional SNPs in *APOBEC3* genes involved in COVID-19 severity, we evaluated the COVID-19 association signals around 7 *APOBEB3* genes, comprising *APOBEC3A*, *APOBEC3B*, *APOBEC3C*, *APOBEC3D*, *APOBEC3F*, *APOBEC3G*, and *APOBEC3H*, in the two COVID-19 hospitalization GWASs with European and African ancestries (HGI-B2-EUR and HGI-B2-AFR, respectively). Around these 7 *APOBEC3* genes, with an arbitrary association threshold of *P* < 0.01, we obtained 2 SNPs from HGI-B2-AFR and 4 SNPs from HGI-B2-EUR. Of these 6 COVID-19 risk SNPs, rs12168809 (*P* = 0.002; OR = 1.12; 95% CI [1.04–1.2]) and rs76929059 (*P* = 0.004; OR = 0.82 [0.71–0.94]) are unique to AA; they are located in the intergenic and promoter region of *APOBEC3A*, respectively. For other 4 SNPs, including rs2076109 (*P* = 0.008; OR = 1.04 [1.01–1.08]), rs1807558 (*P* = 0.01; OR = 1.04 [1.01–1.08]), rs2244104 (*P* = 0.008; OR = 0.96 [0.93–0.99]), and rs13057307 (*P* = 0.009; OR = 1.03 [1.01–1.05]), they are unique to EA (Fig. 1A). It is important to point out that these SNPs are only nominally significant in AA or EA. In conclusion, 2 and 4 prioritized SNPs close to the *APOBEC3* gene cluster were revealed nominally associated with COVID-19 hospitalization in EUR and AFR samples, respectively.

To study whether these SNPs are specific polymorphisms to populations with unique ancestries, we checked the minor allele frequencies of these 6 SNPs in five major populations (including African [AFR], Ad Mixed American [AMR], East Asian [EAS], European [EUR], and South Asian [SAS]) as well as in its corresponding subpopulations (Fig. 1B; Table S1). We revealed that the protective SNP rs76929059 derived from HGI-B2-AFR is only polymorphic in AFR populations [minor allele frequency (MAF) = 0.07]. Another SNP, rs12168809 from HGI-B2-AFR, is a risk SNP to COVID-19 hospitalization and shows higher MAF in AFR as 0.43, compared to all other 4 major populations (mean MAF = 0.19 ± 0.057). In terms of these 4 SNPs emerged from HGI-B2-EUR, the risk SNP rs13057307 is less frequent in AFR (MAF = 0.24) than in other 4 major populations (mean MAF = 0.45 ± 0.09); other 3 SNPs, including rs1807558, rs2076109, and rs2244104, display similar MAFs between AFR (MAFs = 0.28, 0.32, and 0.23, respectively) and other 4 major populations (mean MAFs for the 3 SNPs: 0.30 ± 0.11, 0.38 ± 0.08, and 0.29 ± 0.05, respectively). The latter 3 SNPs were only emerged in HGI-B2-EUR as candidate SNPs and further evaluation revealed that they were not nominally significant in HGI-B2-AFR; given the relative high MAFs for these SNPs across all populations (MAFs ≥ 0.28), if they were associated with COVID-19 hospitalization, they might be expected to show association signals close to the association *P* threshold as *P* < 0.01 in HGI-B2-AFR GWAS, however, we didn’t observe this phenomenon. Therefore, based on this observation, we decided to prioritize only 3 out of 6 SNPs as top candidates, which are rs76929059, rs12168809, and rs13057307, with the first 2 SNPs are more frequent in AFR populations.

Since these 6 SNPs only showing nominal association significance with COVID-19 hospitalization, we wanted to further evaluate their potential involvement in COVID-19 hospitalization based on their potential regulatory roles on *APOBEC3* gene expression. We manually collected cis-eQTL results for these 6 SNPs from GTEx and Haploreg4. Our investigation uncovered all these 6 SNPs, except rs1807558, are nominal significant eQTLs (Fig. 1C). Among the 3 prioritized candidate SNPs, including rs76929059, rs12168809, and rs13057307, the last SNP is highly associated with *APOBEC3C*/*D*/*G* gene expressions across multiple GTEx tissues, with subcutaneous fat and nerve fibers are two tissues where higher correlations between rs13057307 and *APOBEC3* expression were observed (Fig. 1C). While rs12168809 (represented by its high LD SNP rs5757372) is the only eQTL of *APOBEC3A*/C in blood tissue. Another top prioritized SNP rs76929059 (represented by its high LD SNP rs113819742) is an eQTL for multiple *APOBEC3* genes, including *APOBEC3A* (brain caudate basal), *APOBEC3B* (breast mammary tissue, pancreas, brain caudate basal, esophagus muscularis), *APOBEC3C* (colon sigmoid), and *APOBEC3D* (ovary, heart atrial appenda, artery coronary, skin sun exposed low part, esophagus mucosa). In terms of another 3 SNPs that show close MAFs across different major populations, rs1807558 is not an eQTL for all *APOBEC3* genes, rs2076109 is an eQTL for both *APOBEC3B* (cell-cultured fibroblasts and thyroid) and *APOBEC3F* (thyroid); rs2244104 is an eQTL for *APOBEC3C* (muscle skeletal, cells EBV-transformed lymphocytes, lung, adipose subcutaneous, esophagus muscularis, thyroid, nerve tibial, and artery tibial), *APOBEC3D* (adipose subcutaneous), and *APOBEC3F*/*G* (esophagus mucosa). Taken together, our prioritized 3 SNPs, including rs76929059, rs12168809, and rs13057307, are all eQTLs of *APOBEC3* genes.

**Figure 2:**
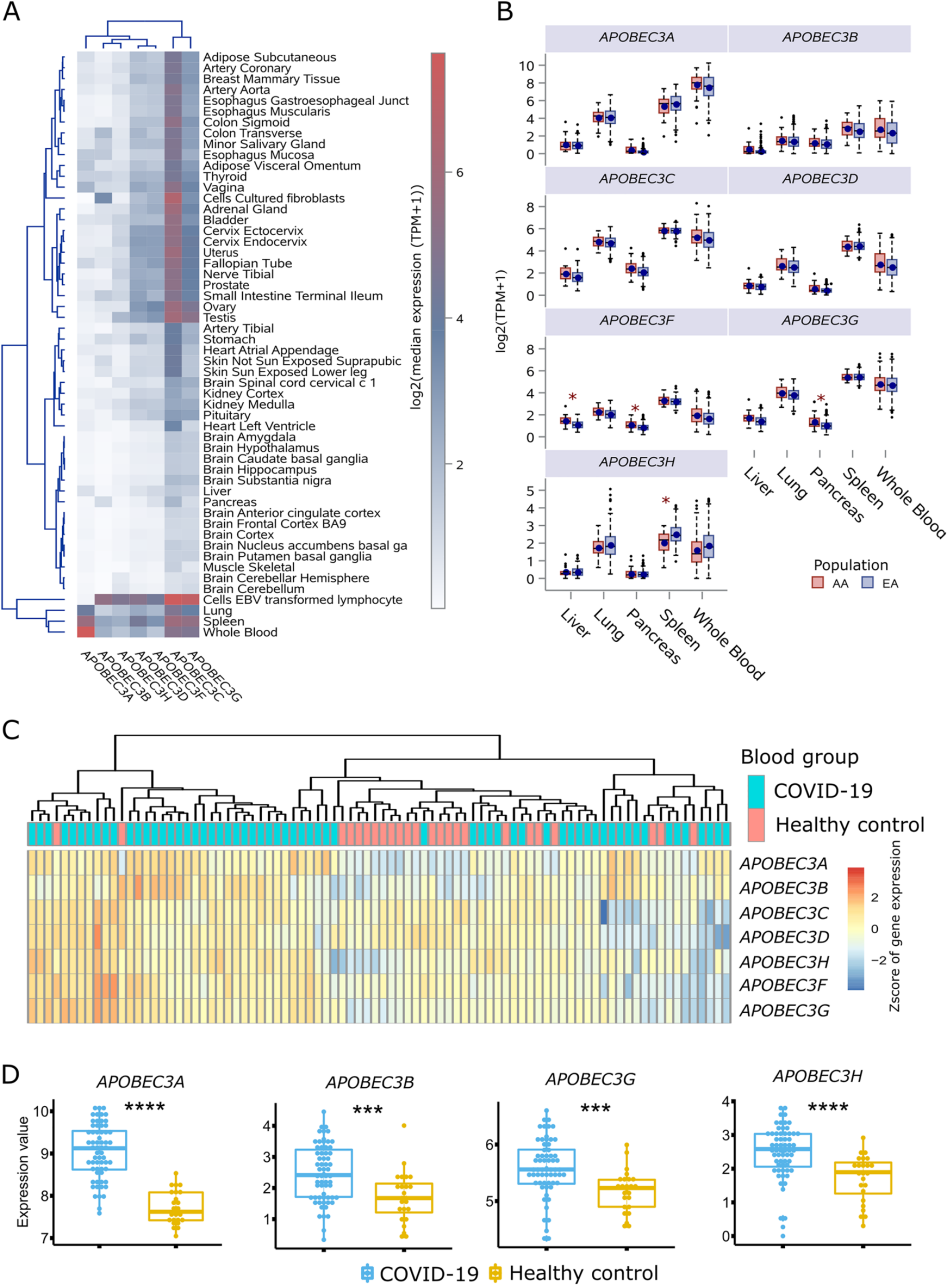
*APOBEC3* gene expression levels across multiple tissues with European or African ancestry in GTEx and their expression levles upon SARS-CoV-2 infection in blood samples from COVID-19 patients. (**A**) Heatmap illustrates the median expression of *APOBEC3* genes across 49 GTEx tissues, with *APOBEC3C*/*G* are highly expressed more than 20 GTEx tissues and other *APOBEC3* genes are moderately expressed in whole blood, spleen, lung, and cells—EBV transformed lymphocyte. (**B**) Boxplots display gene expression levels of *APOBEC3* genes among 5 major GTEx tissues with only 3 *APOBEC3* genes marked by ‘*’ showing significantly differential gene expression between European American (EA) and African American (AA). The statistical significance threshold was set at *P* < 0.00015 after multiple testing adjustment. (**C**,**D**) Clustering analysis and one-way ANOVA analysis of *APOBEC3* genes expression upon SARS-CoV-2 infection in whole blood from healthy controls or COVID-19 patients, with only *APOBEC3A*, *APOBEC3B*, *APOBEC3G*, and *APOBEC3H* are significantly up-regulated after SARS-CoV-2 infection. Note: ***, *P* < 0.001; ****, *P* < 0.0001, and multiple testing *P* threshold was set as *P* < 0.05 / 7 = 0.01.

Additionally, we evaluated *APOBEC3* genes expression in 49 normal tissues by ancestry from GTEx and conducted differential expression analysis for these genes between blood tissues derived from COVID-19 patients and healthy controls. *APOBEC3C*/*G* were highly expressed in > 20 GTEx tissues (median TPM > 2), while other *APOBEC3* genes were only moderately expressed in whole blood, spleen, lung, and cells culture fibroblasts (Fig. 2A). We further performed differential gene expression analysis for these 7 *APOBEC3* genes among 49 GTEx tissues between European American (EA) and African American (AA), and the significance threshold after multiple testing adjustment was set at *P* < 0.00015 = 0.05/(7 × 49). Figure 2B shows the expression profiles of seven *APOBEC3* genes among five major tissues, including liver, lung, pancreas, spleen, and whole blood. Only 3 *APOBEC3* genes, including *APOBEC3F* (in liver and pancreas), *APOBEC3G* (in pancreas), and *APOBEC3H* (in spleen) display significant differential expression. In whole blood, although all 7 *APOBEC3* genes demonstrate nominally significantly (*P* < 0.05) differential gene expression between EA and AA, no ones were survived after multiple testing. Furthermore, we re-analyzed previously published data to determine the expression of *APOBEC3* genes upon SARS-CoV-2 infection in whole blood derived from COVID-19 patients and healthy controls. Cluster analysis and one-way ANOVA analysis of *APOBEC3* gene expression post SARS-CoV-2 infection showed *APOBEC3A*, *APOBEC3B*, *APOBEC3G*, and *APOBEC3H* were significantly upregulated in blood samples from patients with COVID-19 disease compared to healthy controls (Fig. 2C,D). Taken together, *APOBEC3* genes show differential expression profiling across multiple tissues, with *APOBEC3C/G* ubiquitously expressed in more than 20 tissues*,* and 3 tissues, including lung, whole blood, and spleen demonstrate similar expression pattern for these *APOBEC3* genes; *APOBEC3F*/G/H display differential expression levels between EA and AA in specific tissues and *APOBEC3A*/*B/G/H* are upregulated upon SARS-CoV-2 infection in whole blood.

**Figure 3:**
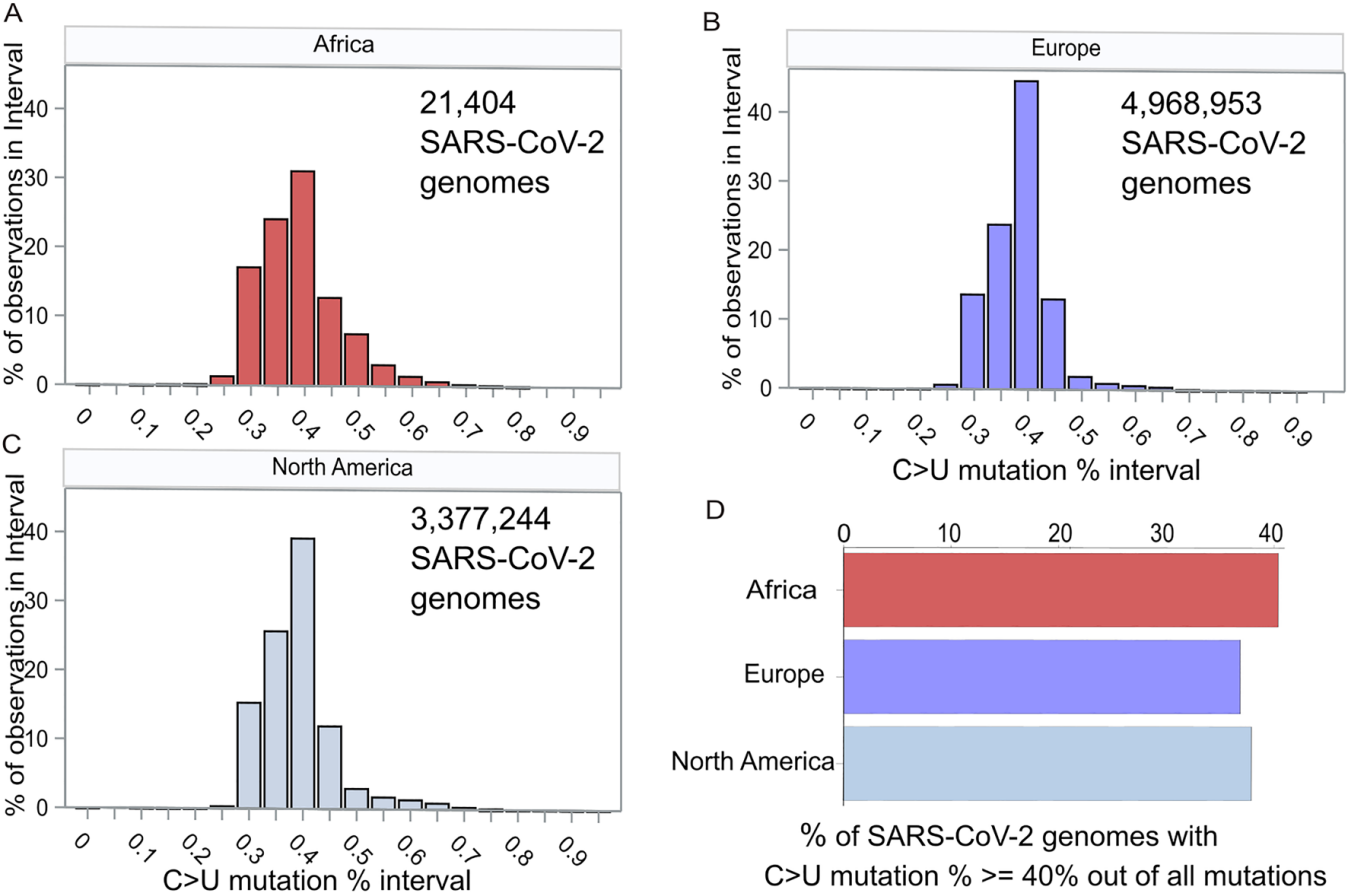
SARS-CoV-2 genomes exhibit a higher percentage of C > U coding mutations in the African region compared to Europe and North America. Histograms depict the distinct distributions of C > U coding mutation percentages across three geographic regions: (**A**) 21,404 SARS-CoV-2 genomes from Africa, (**B**) 4,968,953 SARS-CoV-2 genomes from Europe, and (**C**) 3,377,244 SARS-CoV-2 genomes from North America. (**D**) Notably, the African region-derived SARS-CoV-2 viral genomes display a significantly higher C > U coding mutation percentage when compared to those from Europe and North America (All *P*-values from the Chi-squared test < 1 × 10^–4^).

Given the cumulative studies supporting the involvement of *APOBEC3* genes^25,26^, particularly *APOBEC3A*^27,28^, in the prevalent C>U coding mutations observed in SARS-CoV-2 genomes, we posited that SARS-CoV-2 genomes sampled from African regions would exhibit a higher percentage of C>U coding mutations compared to those sampled from Europe or North America, where the majority of the population shares European ancestry. To validate this hypothesis, we analyzed mutational data from SARS-CoV-2 genomes provided by nextstrain database, comparing the C>U coding mutation percentage per SARS-CoV-2 genome across three geographic regions: Africa, Europe, and North America. The nextstrain database shared open data encompassed 21,404, 4,968,953, and 3,377,244 samples from these respective regions (Fig. 3A–C). In our analysis, which is consistent with prior research^3^, we found that the C>U coding mutation comprised roughly 40% of all coding mutations per SARS-CoV-2 genome. Notably, samples from Africa displayed an increasing percentage of SARS-CoV-2 genomes containing higher C>U coding mutation rates (≥ 40%; Fig. 3D). Furthermore, upon comparing the percentage of SARS-CoV-2 genomes harboring elevated C>U coding mutation rates across the three geographic regions, we observed that Africa exhibited a significantly higher proportion of samples with advanced C>U coding mutation rates (40.38%). In contrast, Europe and North America had lower percentages of SARS-CoV-2 genomes passed the coding mutation rate threshold (C>U mutation percentage ≥ 40%) (Fig. 3D). In summary, through the analysis of approximately 8.4 million SARS-CoV-2 genomes sampled from Africa, Europe, and North America, we have confirmed a significantly higher percentage of SARS-CoV-2 genomes sampled from Africa displaying prominent C>U coding mutation rates.

## Discussion

Previous research has strongly suggested the involvement of *APOBEC3* genes in viral infection through RNA editing, with APOBEC3A was reported to play a critical role in inducing C>U mutation following SARS-CoV-2 infection in vitro^28^. Our exploratory analyses suggested rs12168809 and rs76929059 are located within the intergenic and promoter region of *APOBEC3A*, respectively, and are polymorphic only in AFR populations. These SNPs have also been identified as eQTLs for multiple *APOBEC3* genes across variable tissues, which suggests that they may regulate gene expression levels. Therefore, it is hypothesized that the rs12168809 and rs76929059 SNPs may be responsible for the more frequent emergence of new SARS-CoV-2 variants in AFR populations. The mechanism underlying this association is currently unknown but may involve the regulation of *APOBEC3A* expression levels by rs12168809 and rs76929059 SNPs, which would influence the RNA editing capacity of APOBEC3A. In addition, analyses of over 8.4 million SARS-CoV-2 genomes from Africa, Europe, and North America have enabled the coding mutation rate of C>U to be compared among these continents. Notably, the coding mutation rate of C>U in Africa was found to be significantly higher (40.38%) compared to Europe (36.88%) and North America (37.91%) with *P* values less than 1 × 10^–4^. These observations indicate that the unique genetic makeup in the loci *APOBEC3* genes and the significantly prominent coding mutation rate of C>U of SARS-CoV-2 genomes in AFR population might be the underlying reason why the new SARS-CoV-2 variants are more frequently emerging in AFR, since these *APOBEC3* genes are likely involved in the generation of SARS-CoV-2 mutations.

There were cumulative reports supporting the involvement of *APOBEC3* genes in viral lifecycle. One of the important roles played by APOBEC3 proteins is to direct restrict the virus infection/replication^29^, as all seven APOBEC3 proteins could bind RNA and single strand DNA^30^ to combat retroviruses as well as other pathogenic viruses. APOBEC3A was shown to decrease E2A SUMOylation and interfered with replication of a DNA virus—human adenovirus by deamination^31^. Also, another double strand DNA virus—human papillomaviruses was reported to be edited and inhibited by over expression of APOBEC3A in vitro^32^. Notably, in cells infected by SARS-CoV-2, the introduction of APOBEC3A through exogenous expression led to UC-to-UU mutations in viral RNA, while the expression of other APOBEC proteins did not show the similar effect. Moreover, the mutated C bases were frequently observed at the ends of bulge or loop regions in the secondary structure of the viral RNA^27^. In lines with our finding that whole blood-derived *APOBEC3A*/*B*/*G*/*H* were significantly upregulated after SARS-CoV-2 infection, a recent study suggested SARS-CoV-2 adapts and evolves through APOBEC3A/G and APOBEC1-mediated UC-to-UU mutations in vitro^28^. In terms of APOBEC3B, it was predominately expressed in nuclear that limited its anti-viral spectrum. By examining the samples from EA and AA, one study found heterozygous deletions of *APOBEC3B* had no effect, but homozygous deletions had effect on a direct association with HIV-1 acquisition, progression to AIDS, and viral set points^33^. Likewise, APOBEC3B was reported to deaminate both the negative-sense and positive-sense strand of the para-retrovirus Hepatitis B Virus in vitro and in vivo, resulting in a low proportion of G to A hypermutated viral genome^34^. Furthermore, the upregulated expression of *APOBEC3B* induced by folate deficiency was associated with the inhibition of replication of vesicular stomatitis virus in vitro and in vivo^35^. Recently, APOBEC3B was shown to combine with Poly (A) binding protein cytoplasmic 1 to stimulate protein kinase R (PKR) and overturned the impaired activity of PKR that caused by Sendai virus infection, since stimulation of PKR would shutoff cellular translation thus cutoff viral gene expression^36^. This research hinted APOBEC3B could affect viral infection via not only editing viral genome but also regulating host innate immunity response. Meanwhile, APOBEC3D is expressed in the cytoplasm, and it can hypermutate the HIV-1 genome, thereby playing a role in HIV-1 diversification^37^. APOBEC3G has drawn significant attention for its exceptional intrinsic anti-HIV activity, and it is currently the most extensively studied protein in the human APOBEC3 family. Most *APOBEC3G* variants show high population-specificity^38^. In contrast, APOBEC3F has lower mutagenicity than APOBEC3G and can induce HIV-1 evolution and drug resistance^37,39^. In our study, we demonstrated that a SNP of *APOBEC3A* located in its promoter is only polymorphic in AFR population and also displays suggestive association with COVID-19 hospitalization. Since APOBEC3A is suggested to the key player to contribute the prevalent C>U mutations in SARS-CoV-2 genomes, our mutational analysis of ~ 8.4 million SARS-CoV-2 genomes from Africa, Europe, and North America, supports the potential involvement of APOBEC3A in the more variable mutational profiles of SARS-CoV-2 genes in Africa. Further investigation with experiments conducted at population level is warranted to confirm the role of APOBEC3A in the more prevalent mutation rate of SARS-CoV-2 in AFR population.

In our study, we found 3 prioritized SNPs are eQTLs of multiple *APOBEC3* genes, two of the SNPs are located into regulating area of *APOBEC3A* and are uniquely polymorphic in AFR. These *APOBEC3* genes all show suggestive differential gene expression in blood samples with African ancestry compared to blood samples with European ancestry. We also noted that *APOBEC3A* expression tended to be higher in blood samples with African ancestry. Furthermore, *APOBEC3A*/*B*/*G*/*H* were upregulated upon SARS-CoV-2 infection in blood samples of COVID-19 patients. Finally, we observed that the Africa region-derived SARS-CoV-2 genomes yielded higher C>U coding mutation percentage than that from Europe and North America. Recently, two publications reported that the C>U mutation in SARS-CoV-2 genome is contributed by APOBEC3A^27,28^. Thus, it is warrant for further replication of the association of 3 prioritized *APOBEC3* eQTLs in association with COVID-19 hospitalization and determine it is APOBEC3A but not other APOBEC3 proteins involved in the generation of high transmissible SARS-CoV-2 in AFR populations.

The primary limitation of this study lies in our inability to employ direct experimental methods for assessing whether the C>U coding mutation in SARS-CoV-2 genomes, attributed to APOBEC3A, occurs at a faster rate in Africans compared to Europeans. To address this question, extensive experimentation involving a substantial number of cell lines, such as lymphoblastoid cell lines derived from both European and African populations, would be required. While this avenue holds significant promise for future research, the associated costs and time required for such experiments fall outside the scope of the current study.

### Supplementary Information

Supplementary Table 1.

## Data Availability

All data generated or analyzed during this study are provided with downloadable links in this article, and the analysis codes and intermediate data will be available from the corresponding author (Dr. Zhong-Shan Cheng) upon reasonable request.
