# Supplementary material for: Regulatory variants of APOBEC3 genes potentially associate with COVID-19 severity in populations with African ancestry
Source: Sci Rep. 2023 Dec 17;13:22435. doi: 10.1038/s41598-023-49791-x (PMC10725877; doi:10.1038/s41598-023-49791-x)
Supplement: Supplementary file 1 — Supplementary Table 1. [file 41598_2023_49791_MOESM1_ESM.docx]

**Table S1 Abbreviations for super- and sub-populations from the 1000 Genome Projects**

| **Super-Population** | **Sub-Population** | **Description** |
| --- | --- | --- |
| East Asian (EAS) | CHB | Han Chinese in Beijing, China |
|  | JPT | Japanese in Tokyo, Japan |
|  | CHS | Southern Han Chinese |
|  | CDX | Chinese Dai in Xishuangbanna, China |
|  | KHV | Kinh in Ho Chi Minh City, Vietnam |
| European (EUR) | CEU | Utah Residents (CEPH) with Northern and Western European ancestry |
|  | TSI | Toscani in Italia |
|  | FIN | Finnish in Finland |
|  | GBR | British in England and Scotland |
|  | IBS | Iberian population in Spain |
| African (AFR) | YRI | Yoruba in Ibadan, Nigeria |
|  | LWK | Luhya in Webuye, Kenya |
|  | MAG | Mandinka in The Gambia |
|  | MSL | Mende in Sierra Leone |
|  | ESN | Esan in Nigeria |
|  | ASW | American's of African Ancestry in SW USA |
|  | ACB | African Caribbean in Barbados |
| Ad Mixed American (AMR) | MXL | Mexican Ancestry from Los Angeles USA |
|  | PUR | Puerto Rican from Puerto Rica |
|  | CLM | Colombian from Medellin, Colombia |
|  | PEL | Peruvian from Lima, Peru |
| South Asian (SAS) | GIH | Gujarati Indian from Houston, Texas |
|  | PJL | Punjabi from Lahore, Pakistan |
|  | BEB | Bengali from Bangladesh |
|  | STU | Sri Lankan Tamil from the UK |
|  | ITU | Indian Telugu from the UK |
